# MOUSSE: Multi-Omics Using Subject-Specific SignaturEs

**DOI:** 10.3390/cancers13143423

**Published:** 2021-07-08

**Authors:** Giuseppe Fiorentino, Roberto Visintainer, Enrico Domenici, Mario Lauria, Luca Marchetti

**Affiliations:** 1 Fondazione The Microsoft Research, University of Trento Centre for Computational and Systems Biology (COSBI), 38068 Rovereto, Italy; fiorentino@cosbi.eu (G.F.); visintainer@cosbi.eu (R.V.); domenici@cosbi.eu (E.D.); lauria@cosbi.eu (M.L.); 2 Department of Cellular, Computational, and Integrative Biology (CiBio), University of Trento, 38123 Povo, Italy; 3 Department of Mathematics, University of Trento, 38123 Povo, Italy

**Keywords:** multi-omics data integration, precision medicine, biomarker identification, unsupervised clustering, cancer

## Abstract

**Simple Summary:**

Modern profiling technologies have led to relevant progress toward precision medicine and disease management. A new trend in patient classification is to integrate multiple data types for the same subjects to increase the chance of identifying meaningful phenotype groups. However, these methodologies are still in their infancy, with their performance varying widely depending on the biological conditions analyzed. We developed MOUSSE, a new unsupervised and normalization-free tool for multi-omics integration able to maintain good clustering performance across a wide range of omics data. We verified its efficiency in clustering patients based on survival for ten different cancer types. The results we obtained show a higher average score in classification performance than ten other state-of-the-art algorithms. We have further validated the method by identifying a list of biological features potentially involved in patient survival, finding a high degree of concordance with the literature.

**Abstract:**

High-throughput technologies make it possible to produce a large amount of data representing different biological layers, examples of which are genomics, proteomics, metabolomics and transcriptomics. Omics data have been individually investigated to understand the molecular bases of various diseases, but this may not be sufficient to fully capture the molecular mechanisms and the multilayer regulatory processes underlying complex diseases, especially cancer. To overcome this problem, several multi-omics integration methods have been introduced but a commonly agreed standard of analysis is still lacking. In this paper, we present MOUSSE, a novel normalization-free pipeline for unsupervised multi-omics integration. The main innovations are the use of rank-based subject-specific signatures and the use of such signatures to derive subject similarity networks. A separate similarity network was derived for each omics, and the resulting networks were then carefully merged in a way that considered their informative content. We applied it to analyze survival in ten different types of cancer. We produced a meaningful clusterization of the subjects and obtained a higher average classification score than ten state-of-the-art algorithms tested on the same data. As further validation, we extracted from the subject-specific signatures a list of relevant features used for the clusterization and investigated their biological role in survival. We were able to verify that, according to the literature, these features are highly involved in cancer progression and differential survival.

## 1. Introduction

In the last decade, the advances in high-throughput technologies and the decrease in their cost and throughput time have allowed for extensive production of information at different layers of the biological processes, from the genome down to the metabolome level [1]. The ensemble of information concerning these biological layers is often referred to with the term “omics”, by itself or as a suffix (i.e., transcriptomics, proteomics, metabolomics, etc.). The availability of such data has also led to the development of dedicated repositories, e.g., GEO (Gene Expression Omnibus), TCGA (The Cancer Genome Atlas) [2,3] or cBioPortal, which store multiple datasets covering various diseases, allowing users to easily access and analyze them. Data availability has made possible an increase in the number of bioinformatic investigations on the molecular basis of diseases, the identification of putative biomarkers using different omics and the development of different integrative data analysis techniques [4]. In particular, the possibility of obtaining multiple omics from the same sample has dramatically increased the chances to better understand the regulative processes underlying an observed phenotype by identifying relationships among data layers and strengthening shared signals [5]. Overall, the trend has been in the direction of an increase in the size of datasets and in the complexity of the employed algorithms to carry out multi-omics data analysis [1,6,7,8].

Over the last few years, multiple algorithmic approaches have been proposed to perform multi-omics analysis (e.g., iCluster+ [9], Jive [10], SNF [11], etc.). An open problem resulting from this heterogeneity of computational approaches is the large variability of their performance across different diseases or sets of omics. In order to achieve reliable results, such algorithms often require a fine-tuning of parameters and some careful data preprocessing. These steps are often difficult to select a priori, thus limiting the ease of use and applicability of the tools. For all these reasons, a gold standard for multi-omics analysis workflow is still missing, as shown by previous comparative analyses [12,13]. As a result, the impact of multi-omics integration on disease knowledge is still lagging behind the single omics analysis. In an effort to address these shortcomings, we herein introduce MOUSSE, a novel normalization-free pipeline for unsupervised multi-omics integration able to obtain a higher average classification score than ten state-of-the-art algorithms in analyzing patient survival in ten different types of cancer.

The method is based on replacing the whole patient profile with a rank-based subject-specific signature. This has different advantages, such as dimensionality reduction and increased robustness to measurement noise or data quality issues. This approach demonstrated its efficacy in a previous single omics algorithm, SCUDO (Signature-based ClUstering for DiagnOstic purposes) [14], which ranked second overall in the SBV Improver Diagnostic Signature Challenge [15], and which has been successfully used to analyze single omics datasets in multiple biological contexts [16,17,18,19,20,21,22]. With respect to SCUDO, our novel method extends the use of patient-specific signatures to provide a complete multi-omics pipeline. As part of the design, we employed additional principles borrowed from information theory, such as the Kullback–Leibler divergence [23] (DKL), and a methodology centered on a transformation-based integration relying on tailored subjects’ similarity networks. The first principle is optionally used to select signature lengths automatically, whilst the latter is used to fuse different types of data through a common intermediate form, the similarity network, calculated through a highly sensitive measure, the rank-biased overlap [24].

We verified the variation in the performance of our tool by testing it on ten different TCGA datasets, including methylome, transcriptome and miRNAome data for every subject. We used the same benchmark datasets of previously published reviews, including a comparative evaluation of multiple state-of-the-art integration algorithms [12]. We performed a survival analysis of the obtained clusters, following the benchmark protocol, and compared our results with ten state-of-the-art algorithms. We achieved the highest median performance, confirming the stability of our pipeline. To verify if our clusters were biologically meaningful, we then investigated the signatures computed by our method and obtained a high concordance with previous findings from the literature.

## 2. Results

**Figure 1:**
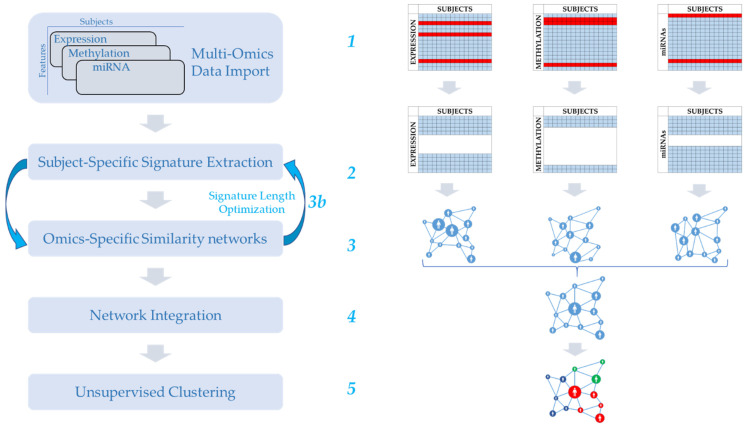
Graphical representation of the MOUSSE methodology (see Materials and Methods for details). (**1**) Omics datasets have to be provided in the form of matrices sharing the same set of subjects (in our analysis, data were downloaded from TCGA). (**2**) Input is converted into ranked subject-specific lists and then reduced to produce the signatures. (**3**) For each omics, the subjects are mapped into a network based on signature similarity. (**3b**) Optional optimization step in order to select the best signature length by calculating and comparing the networks built from signatures of different lengths. (**4**) The networks are fused into one. (**5**) Clusters are identified in an unsupervised manner from the joint network.

To achieve top-level performances, we designed the MOUSSE pipeline by leveraging a wide range of original computational approaches to process and transform our input data (see Figure 1). For a more detailed explanation of their use and additional technical details, we refer to the Discussion and Materials and Methods.

### 2.1. Benchmark vs. State-of-the-Art Software

To effectively verify the tool’s performance and its reliability in different conditions, we decided to test it on multiple types of cancer, a highly heterogeneous and complex disorder. We used a set of ten multi-omics datasets from TCGA, which were used in a previously published benchmark [12] to test our performance versus ten multi-omics algorithms representing the current state of the art. In particular, we included: K-means [25], Spectral Clustering [26,27], LRAcluster [28], PINS [29], rMKL-LPP [30], MCCA [31], MultiNMF [32], iClusterBayes [33], SNF [11] and NeMo [34].

**Figure 2:**
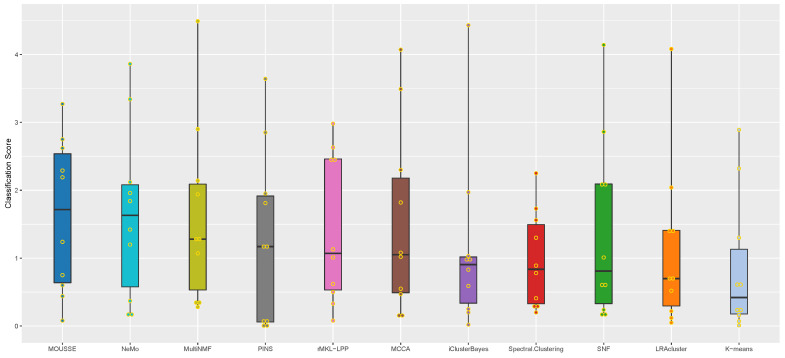
Box-and-whisker plot of classification score values for all the tools analyzed across the ten cancer datasets considered in the benchmark. The algorithms are sorted from left to right in decreasing order of their median classification score.

For each considered multi-omics dataset, we applied the algorithms to group the patients included in the study into clusters representing different degrees of survival. We then present the classification score of each algorithm by identifying clusters of subjects displaying different average survival times (see Materials and Methods for details). Survival analysis is a common validation methodology for unsupervised algorithms due to the absence of a clear patient classification for many cancers and the large variation in clinical parameters. In Figure 2, we compare the performance of MOUSSE against the performance of the other tools, as reported in Rappoport et al. [12] using a box-and-whisker plot of classification score values across the ten cancer datasets; in Table 1 we summarize the results of this performance comparison. The clusters obtained for each cancer and the Kaplan–Meier curves associated are reported in Figures S1–S10.

Due to the extreme differences in the biology of cancers of different tissues, achieving the best survival-based clustering in all datasets is a very challenging task, as indicated by the relatively low overall average performances reported in Table 1.

**Figure 3:**
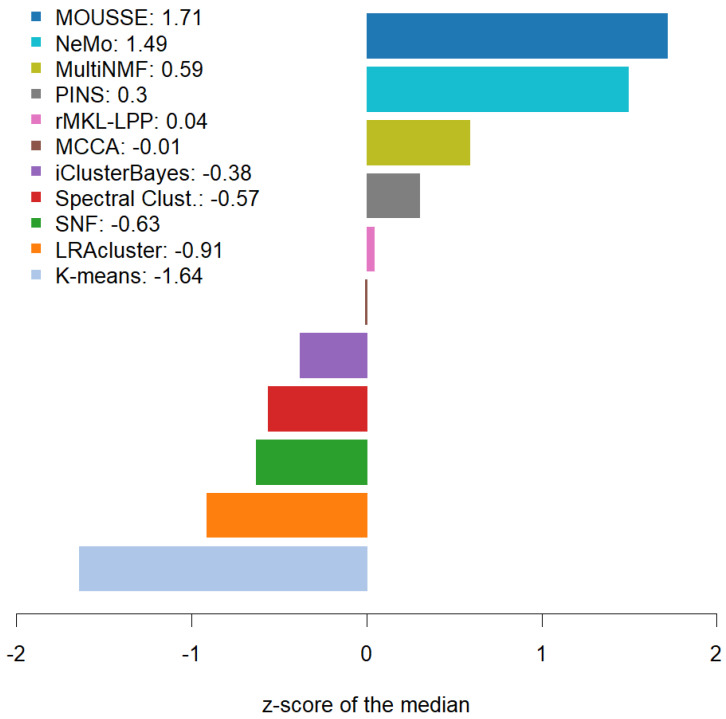
Bar plot of the z-scores calculated on the classification score medians achieved by the software across the ten cancer types.

However, our tool was able to achieve the highest median performance across the ten cancers and the best z-scores. MOUSSE has a median classification score that is almost two standard deviations (+1.71 z-score) higher than the average value, achieving a slightly higher score than the second-best method, NeMo (+1.49). These two methods performed similarly well, being almost one standard deviation above the third (MultiNMF = 0.59 z-score) and more than three from the lowest scoring one (−1.64, see Figure 3). However, MOUSSE has a lower performance variability and a higher upper quantile (Figure 2). Differently from NeMo, MOUSSE can readily produce an informative biomarker as a result of its computation, as shown in the following section.

### 2.2. Biological Validation

A useful feature of MOUSSE is that the user can leverage the subject-specific signatures automatically computed by the pipeline to extract a biomarker underlying the identified patient clusters. This allows using the pipeline not only to stratify patients but also to suggest important biological actors that can be further analyzed to provide new insights on the considered cancers. To further validate this important feature of the pipeline, we analyzed the biomarkers extracted from the three cancer types for which MOUSSE provided the best classification performance based on survival analysis (BIC, GBM, and LIHC). To maximize the strength of the experimental evidence in our analysis, we extracted the biomarkers by considering the subjects from the two identified clusters with the highest and lowest mean survival. The features included in the biomarkers were taken from the subjects’ specific signatures, filtered using a Wilcoxon test with Bonferroni correction (corrected *p*-value < 0.05). The full list of all the significant features extracted for each cancer and their *p*-value is available in Material S2. We then searched the current literature to see if the features extracted from each omics were known to be involved in the development of the investigated cancer. Furthermore, when possible, we verified if their differential expression in our clusters was consistent with the literature. The methylation data that we integrated contain many methylation probes which are hard to associate with differential expression. Therefore, we focused our analysis on the miRNAs and gene signatures (Table 2).

According to the current literature, 86% of the miRNAs and a third of the genes identified by MOUSSE are highly involved in cancer progression and/or prognosis (in green in Table 2). The directionality of differential expression (defined as the sign of the logarithm of fold change) is consistent with results reported in the literature regarding survival, with no exception. A list of the literature supporting the biomarker and its involvement in cancer development and/or prognosis can be found in Tables S1 and S2, while some of the most relevant will be discussed later. The high presence of features related to differential survival further supports the reliability of the clusterization computed by MOUSSE and highlights the potential of our pipeline as a tool to investigate biological processes and suggest novel actors underlying the observed phenomenon.

## 3. Discussion

We focus our discussion on the novel aspects of the pipeline and the analysis of the benchmark results.

### 3.1. Pipeline Novelties

We present a reliable multi-omics analysis tool that is easy to use and that can be potentially used with any kind of omics data and easily adapted to a wide range of applications. The modularity of the produced pipeline makes it possible to change any component block with an updated or customized implementation without loss of functionality.

One of the main problems encountered when handling different kinds of biological data together is the wide range of variability that different datasets of multiple omics can have [4]. This may include a different scale of measurements or ranges. To overcome this, we decided to rely on a rank-based signature approach. This methodology allows for the reduction in the size of the input profile of each subject into a smaller subset, here referred to as a signature, representing the list of the most and least expressed features within the profile (i.e., the features with the highest and the lowest ranks). The use of ranks reduces the impact of the heterogeneity of the omics data and allows for the development of a normalization-free method. This approach has been proven to maintain the information necessary to characterize the clusters, despite reducing the size of the input datasets, as shown by our current results and by previous studies [16,17,18,19,20,21,22]. While the dataset reduction has very often a positive impact on the total runtime, it may lead to loss of important information, especially if the dataset noise is extremely high or the arbitrary selection of the signature length is not ideal. For this reason, we inserted an optional optimization strategy to identify the signature length and we also allowed the user to provide a manual selection. While the optimization strategy automatically selects the signature that, according to the Kullback–Leibler Divergence, has the highest information, the manual selection is to be preferred if additional biological information is available, or when working on a characterized short set of biological entities (genes, miRNAs, proteins, etc.).

The use of short subject-specific signatures allows the user to focus on the most important properties of each patient profile but has the drawback of increasing the number of non-shared features between the signatures. Such a low overlap and the importance of considering the features ranks during the comparison prevents the use of standard metrics such as Euclidean distance or edit distance for our analysis. For this reason, our pipeline is based on RBO [24], a similarity measure created to compare ranked lists. Using this kind of measure, we were able to selectively weigh overlapping and non-overlapping features depending on their ranks, thus building a more reliable similarity network. When using data of different omics, datasets with a low number of variables or signatures with low intra-subject variability, other metrics may be more suitable. For this reason, we left the possibility in MOUSSE to switch to a different similarity measure to produce the similarity matrix, if needed. It is also possible to change our default clustering algorithm, which is currently set to spectral clustering, having shown its reliability in multiple biological applications [35,36,37]. The combined effect of these design choices grants MOUSSE a rather stable performance across different datasets. It is interesting to note that MOUSSE, NeMo and SNF share a general architecture that relies on similarity networks. However, while MOUSSE and NeMo obtain a similar average performance and result in the two most reliable algorithms, SNF achieves a considerably lower median performance. MOUSSE and NeMo build their similarity networks using a more advanced metric than the Euclidean distance employed by SNF. Since MOUSSE utilizes the same integration as SNF, the resulting differences in performance suggest that the method used for the construction of the similarity network is crucial. However, it is hard to precisely decouple the contribution of the similarity network construction and the integration method. When we tried to integrate our omics-specific similarity network with an average-based method, similarly to NeMo or using the SNFtools “Similarity Matrix” function to convert the distance to a similarity, we obtained a lower performance.

### 3.2. Benchmark Results

Our results and previous comparisons [12,34] show how all the investigated multi-omics integrating tools are unable to maintain the uniform quality of their clustering performance when applied to multiple cancers. No single method appears to consistently perform better than the others; this fact is observable in Figure S11, which shows the relative performance of each tool across the ten types of cancer in the dataset. In this context, the performance advantage of MOUSSE derives from the robustness of its classification performance, consistently achieving an above-average ranking across the majority of the datasets.

MOUSSE hits the top spot in three out of ten datasets. This is in contrast with almost all of the other algorithms, which achieve top performance in single studies, typically the one considered in the original publication. When applied to multiple cancers, the average result is considerably lower (e.g., SNF in GBM = 4.136, mean across cancers = 1.4), highlighting the importance of a sufficiently large and diverse collection of datasets in carrying out a thorough performance evaluation. This effect could be due to the high heterogeneity between different types of cancers, affecting completely different tissues or organ systems. One practical consequence of this observation is that the method that is going to perform best on a specific cancer is very dependent on the nature of the data, and there is currently no way to predict a priori the winning method, given a dataset. Intriguingly, some of the methods have highly complementary performance profiles, as shown in Figure S11. For example, rMKL-LPP appears to reach the top rank with the COAD and SARC datasets, for which MOUSSE achieves its worst performance; the roles are exactly reversed for the BIC, KIRK and LUSK datasets. This observation suggests a possible path forward for the design of the next generation of multi-omics analysis algorithms, in which sufficiently diverse methods are combined into a single ensemble method. It was shown previously [38,39] that by combining predictions from different classifiers, the quality and robustness of predictions can be improved. The crucial issues to be solved to implement this type of design are the selection of a set of algorithms with sufficiently complementary classification outcomes (such as those of rMKL-LPP and MOUSSE), and the details on how to combine the individual outputs to obtain a single outcome.

The use of a benchmark on multiple datasets also allowed us to identify the most difficult datasets for multi-omics analysis, as shown by the drop of absolute classification score values in COAD, LUSC and OV, shared by all the algorithms. We were not able to ascertain whether this underperformance was caused by a higher biological complexity or by data quality, or a combination of both, but future studies may shed some light on the matter and possibly suggest a remedy. Even suffering this performance drop, MOUSSE was able to maintain the most stable performance across the ten cancer types, suggesting higher consistency of results if applied to a wider range of diseases.

To further confirm the validity of our methodology, especially on the use of the subject-specific signatures, we looked at the biological information carried by the biomarker features extracted from the BIC, GBM and LIHC datasets. In each cancer, we focused on the clusters with the highest difference in survival to investigate differentially expressed features. As expected, the extracted biomarker, shown in Table 2, contained features involved in prognostic changes or with a relevant impact within cancer: 46 out of 60 features were reported to be altered in their respective cancer literature, 35 of which were strictly linked to cancer severity, heavily impacting its development, and thus influencing survival. Furthermore, when looking at low and high prognosis clusters, we always found the same directionality in our data and literature. All the identified significant miRNAs have been reported in regulatory mechanisms involved in cancer development, by directly targeting genes (e.g., mir-760 [40], mir-106a [41], mir-214 [42], mir-199b [43]) or by working in a complex with other miRNAs (e.g., mir-105-1/mir105-2/mir767 [44], mir-221/222 [45]) or even by interacting with other classes of RNAs (e.g., mir-324 is sponged by a long non-coding RNA [46]). Furthermore, some of the miRNAs have high relevance for treatment due to their ability to target multiple genes and pathways (e.g., mir-204 [47,48,49], mir-340 [50]) or because they influence treatment resistance, both chemical and radiotherapeutic (e.g., mir-760 [51] with doxorubicin mir-130b [52]). Four miRNAs have already been reported as potential prognostic biomarkers (mir-221 [45], mir-222 [45], mir-301 [53], mir-199a [54]). Similarly, among the relevant genes, TPX2 [55], SEC61G [56], CRTAC1 [57], SLC11A1 [58] and EXO1 [59] have been suggested as prognostic biomarkers and/or potential therapeutic targets. In a GBM deep learning model, with a concordance index of ≈0.70, PLA2G2A [60] was one of the 10 most relevant genes for predicting survival. SEC61G [56], NEK2 [61] and DDX53 [62] are known to alter the response to chemicals (e.g., sec61g/temozolomide) or radiotherapies, thus influencing survival. Interestingly, MOUSSE’s biomarker suggests potential targets for future investigations. For example, both NEK2 and the mir-105-1/105-2/767 miRNAs complexes are found in the LIHC biomarker. These nucleic acids share the effect on sorafenib resistance, a kinase inhibitor approved as a treatment in multiple cancers. The mechanisms underlying this resistance are still unknown, thus an interaction between the gene and the miRNA should be investigated in the future. COL16A1 has been found to reduce glioma invasiveness [63], and while we could not find a study that associated it with survival, an automatized survival analysis reported by The Human Protein Atlas suggests a link to differential survival in glioma (https://www.proteinatlas.org/ENSG00000084636-COL16A1/pathology; accessed on 2 July 2021). The transcription factor TOX3 [64] is not associated with GBM survival but was found to be co-expressed with Nestin and can bind its promoter. The involvement of Nestin in GBM and its use as a biomarker for glioma stem cells [65] suggests a possible interaction of TOX3 in this cancer. Similarly, GPR17 is linked to an altered pathway in glioblastoma and its targeting has led to a significant reduction in neurospheres in rodent models [66]. These cellular structures influence glioblastoma aggressiveness and are used as clinical predictors [67].

To investigate the diseases and therapeutic areas that could be linked to our biomarkers, we used the 200 most relevant genes identified for BIC, GBM and LIHC in OpenTargets (https://www.targetvalidation.org/; accessed on 2 July 2021). Even if our biomarker is an extremely small fraction of the original dataset (200/≈18800), we always found cell proliferation disorder as the first therapeutic area, with at least 120/200 genes involved. We also found an extremely high association with cancer (min 115/200 genes involved), neoplasm (min 118/200), carcinoma (min 99/200) and organ-specific diseases even when reducing the biomarker to the top 50 genes (Supplementary Material 3).

The enrichment in the biomarkers of genes and miRNAs associated with cancer development supports the capability of MOUSSE in identifying biologically meaningful clusters. It also shows the power of this methodology to investigate the regulative processes behind the development of the identified phenotype classes.

While encouraged by MOUSSE’s positive results, we are well aware that both the comparative analysis carried out in this study and the pipeline itself have some limitations. First, the absence of ground truth to verify the correctness of the subjects’ classification has led both us and the authors of the previous papers to use survival as the classification phenotype. This information, especially for TCGA data, is often incomplete or imprecise, due to the lack of complete follow-up for a subset of the subjects, complicating the accurate performance assessment of the algorithms. Second, while we strived to include a large assortment of cancer types, our collection of datasets does not fully represent the biological heterogeneity of human diseases, and thus it is hard to predict how our and other algorithms would fare on an entirely different set of data. Regarding the limitations of the proposed pipeline, one potential shortcoming of MOUSSE is its reliance on the extremal value of each profile. Therefore, it is possible in principle that some types of low-quality data might interfere with the signature selection and have a disproportionate impact on the classification outcome due to the resulting aberrant profile reduction. Furthermore, even if it has a positive impact on the clustering performance, the use of RBO as a similarity measure is computationally demanding. Indeed, the running time of MOUSSE grows more than linearly both with signature length and the number of subjects.

In the future, we aim to refine our analysis by improving the quality of the input data. In our tests, we used a previous benchmark dataset [12,34] based on TCGA data to compare our results with as many state-of-the-art algorithms as possible. Our choice was driven by the lack of literature on multi-omics benchmarking and data standardization. Therefore, we did not investigate confounding variables and used the overall survival for all the cancer types, following the same approach employed in the original analysis. This choice, together with the unsupervised setting, was shared by all the multi-omics tools considered for the benchmark, ensuring a neutral ground for the performance comparisons. However, we are aware of studies that have shown how overall survival is not always reliable, due to the quality of the data themself, and that different survival time variables (e.g., disease-free survival) can improve the survival analysis performances depending on the type of cancer [68]. We aim to take advantage of this knowledge in future work to provide a better survival-based validation, together with improved cluster analysis, possibly accounting for the presence of confounding variables (e.g., sex, age, ethnicity, follow-ups, etc.).

## 4. Materials and Methods

### 4.1. Data Availability

All the raw data for the analysis and the software performances were taken from previous multi-omics reviews [12,34] and can be found at http://acgt.cs.tau.ac.il/multi_omic_benchmark/download.html (accessed on 2 July 2021). 

We utilized the revised data for MCCA and LRACluster (http://acgt.cs.tau.ac.il/multi_omic_benchmark/silhouette_error.html; accessed on 2 July 2021)). The data are composed of ten level 3 TCGA datasets, covering different cancers:

- AML (acute myeloid leukemia);
- BIC (breast invasive carcinoma);
- COAD (colon adenocarcinoma);
- GBM (glioblastoma multiform);
- KIRC (kidney renal clear cell carcinoma);
- LIHC (liver hepatocellular carcinoma);
- LUSC (lung squamous cell carcinoma);
- SKCM (skin cutaneous melanoma);
- SARC (sarcoma);
- OV (ovarian serous cystadenocarcinoma).

Each dataset contains transcriptome, methylome and miRNAome data for each subject. The number of subjects varies across the studies from 170 in AML to 620 in BIC. Cluster validation was carried out using the overall survival time (OS) of the subjects as the benchmark. Similarly, subjects with primary tumors were selected for all cancer types but AML, which included non-solid samples. 

### 4.2. Pipeline Methodology

The technical details of MOUSSE are reported in the following subsection. All the analyses were implemented in R software, using RStudio as a visualizer. A more detailed description of the pipeline is provided in Supplementary Material 1.

#### 4.2.1. Preprocessing

First, all the data underwent the same early preprocessing suggested for all the tools in the Rappoport et al. benchmark [12,34]. We then performed the same preprocessing used for SNF in the review: removal of miRNAs with zero variance, expression data log-transformed and all the features were normalized to have mean 0 and standard deviation 1. Then, features with a coefficient of variation $CVar=\frac{\sigma }{\mu }$ [13] lower than the 5th percentile were removed, to maintain only features with a minimum of variation across subjects.

#### 4.2.2. Subject-Specific Signature Extraction

To extract the signatures, the features of each subject were sorted decreasingly and substituted with their rank. The extraction was then performed by selecting the *n*_1_ most expressed and the *n*_2_ least expressed features (e.g., genes, miRNAs, methylation probes) of each subject. Both *n*_1_ and *n*_2_ can be arbitrarily set depending on the biological application and case specifics. This procedure was repeated for each omics and cancer. 

To reduce the arbitrary choices in our survival analysis, we developed an optimization to automatically select the best value for *n*_1_ and *n*_2_ among a predefined range of signature lengths, as later explained. The values used for our analysis are reported in Table S3. 

#### 4.2.3. Omics-Specific Similarity Networks

For each omics, we used the rank-biased overlap (RBO) [24] to obtain a similarity matrix:

(1) $$RBO\left(S,T,p,k\right)=\frac{X_{K}}{k}*p^{k}+\frac{1-p}{p}\overset{k}{\underset{d=1}{\sum }}\frac{X_{d}}{d}*p^{d}$$

Briefly, this metric measures the similarity between two lists (*S*, *T*) of length *k*, by calculating the overlap at a certain depth ($X_{d}$) and adjusting it through the *p* parameter to give higher relevance to changes at the top of the lists. This parameter depends both on the list length and on a weight that determines how much significance to give to a percentage of the list (set to 0.8 in our implementation). In our analysis, *p* was automatically set to maintain a stable weight, given the changes in the length of the signatures.

Starting from the signatures of length *n*_1_ and *n*_2_ extracted in the previous step (Section 4.2.2), we calculated a pair of similarity matrices by computing the RBO between all the possible pairs of subjects. The average between these two matrices was used as the similarity matrix for one omics when the optimization described in Section 4.2.4 was not used. We considered the omics similarity matrix as the adjacency matrix of the network between the subjects, where each similarity measurement corresponds to the weight of the link between two subjects. To reduce the computational time of the similarity, in the analysis the R package “parallel” was used.

#### 4.2.4. Signature Length Optimization

To automatically select the best signature length for each omics, we relied on the Kullback–Leibler divergence [23], an information theory method that can be applied to measure the information gain obtained by using a distribution (*Q*) to approximate another (*P*).

(2) $$DKL\;(P||Q)=\underset{i}{\sum }P\left(i\right)\;\log_{2}\left(\frac{P\left(i\right)}{Q\left(i\right)}\right)$$

To select the best length, we used a set of different lengths for *n*_1_ in each omics. We produced a similarity matrix for each length chosen. We then calculated the distribution of the values of the similarity matrices computed for each *n*_1_ value. We added a pseudo count of one to each bin and normalized the distribution by dividing it by the area of the distribution. We then calculated the DKL between the shortest signature (*Q*) and each one of the others (*P*) to evaluate the information gained with an increase in length. We chose the length where DKL first reached its maximum value, corresponding to the highest information/noise ratio, and set it as the final *n*_1_. It can be appreciated from Table S4 that the longest signature was not always the most informative. We repeated the same procedure for *n*_2_.

#### 4.2.5. Network Integration and Clustering

The networks resulting from each omics were fused into a single one using the “SNFtools” R package. A single network for each cancer was thus produced. Subjects were then divided into groups using the spectral clustering function of the same package. 

### 4.3. Survival Analysis and Benchmark Comparison

To obtain comparable results and evaluate our method against the other tools tested by Rappoport et al. [12,34], we validated our clustering using the overall survival time and overall survival of the subjects.

For each cancer, we calculated a log-rank curve and compared the survival between the clusters using the “SurvDiff” function of the R package “Survival”. We extracted an empirical *p*-value using the chi-squared statistics as described in Rappoport et al. [12,34]. We then permuted the cluster labels 30,000 times to compute permuted *p*-values. The number of permutations used was different from the one used in the original benchmark; however, we verified that our setting would not significantly affect the score (less than 0.01% variation). A final score, addressed as the classification score, was extracted using the following formula:

(3) $$classification\;score:\;-\log_{10}\left(\frac{number\;of\;times\;cluster^{\prime }s\;p-value\;<\;permutated\;p-values}{number\;of\;permutations}\right)$$

Our results were compared to the classification scores obtained by the other algorithms reported by Rappoport [12,34]. To further evaluate the performance, we extracted the median, the median absolute deviation and the z-score of the median of the results.

### 4.4. Biological Validation

To explore the biology underlying our clustering, we studied the signatures of the two clusters with the highest survival difference in three cancer types and extracted a biomarker of relevant features. To do so, first, we normalized the signatures’ ranks to account for the difference in length between the most and least expressed ($normalized\left(x\right)=\frac{\left(x-\min\left(x\right)\right)}{\max\left(x-\min\left(x\right)\right)}$). We then reversed the obtained 0–1 score by subtracting 1 and divided the values for the number of subjects in the cancer dataset, to account for their frequency. We then fused the most and least expressed signatures after normalization, sorted all the features by their rank-sum and extracted the highest hundred. We then filtered the features, selecting only those that would be significantly different between the two clusters according to Wilcoxon test (R basic function). We adjusted the extracted *p*-value using the Bonferroni correction and selected only the significant results.

To investigate the enrichment of association with diseases in our biomarker, we used OpenTargets [69]. The biomarker used for this analysis was extracted using all the subjects of one cancer and the normalized rank-sum described above without Wilcoxon filtering. The top 50/100/200 genes were then used for the analysis.

## 5. Conclusions

In this paper, we presented MOUSSE, a novel normalization-free pipeline for unsupervised multi-omics integration based on subject-specific signatures. We tested the reliability of our method by analyzing multiple cancer datasets and comparing our performances with 10 other integration algorithms. Survival analysis of the clustering showed the ability of MOUSSE to maintain good performance across a wide range of cancer types and its achievement of the best median score. Furthermore, we extracted and analyzed the biomarkers from the three cancer types for which MOUSSE provided the best classification performance based on survival analysis. The high presence of features involved in cancer development and prognosis, as seen in the literature, supports the value of the pipeline as a tool to also investigate the regulative processes that are behind the development of the identified cancer phenotypes.

## Figures and Tables

**Table 1:**
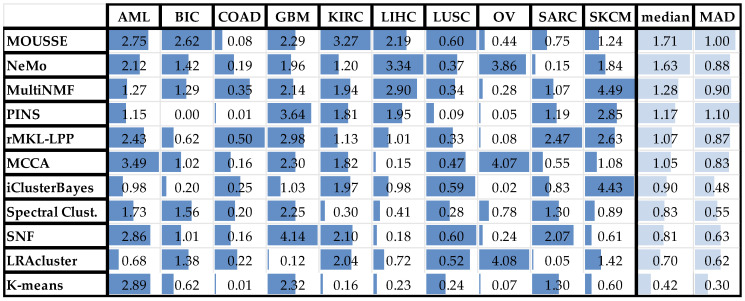
Classification score results across the ten cancers considered in the benchmark. The results are expressed as log10 *p*-value of the survival analysis. The blue bars are a graphical representation of the results normalized by the maximum value of each column. The last two columns report the median and the median absolute deviation (MAD) obtained by each tool.

**Table 2:** List of the 10 most significant features (miRNAs on the left, genes on the right) extracted from the MOUSSE signatures of the selected cancers (BIC: Breast Invasive Carcinoma; GBM: Glioblastoma Multiform, LIHC: Liver Hepatocellular carcinoma). Features with a green background are those already reported in the literature to have a strong influence on prognosis in the selected cancer, especially as prognostic predictors. The directionality (defined as the sign of the logarithm of fold change) of their expression is the same in literature and our biomarker. Yellow background instead identifies genes that have been as-sociated with their cancer but without a quantitative analysis of their expression or a link to survival, thus without directionality information.

miRNA			Genes		
BIC	GBM	LIHC	BIC	GBM	LIHC
let-7c	miR-222	mir-105-2	LOC728264	TOX3	DSCR4
mir-140	miR-23a	mir-767	SLC7A3	SEC61G	SSX6
mir-1307	miR-204	mir-105-1	HSPD1	C20orf42	EXO1
mir-101-2	miR-34b	mir-139	IGFN1	PLA2G2A	NEK2
mir-33b	miR-221	mir-199a-1	AURKA	CRTAC1	RHOXF2B
mir-99b	miR-340	mir-199a-2	ANGPTL7	CA10	DCAF8L1
mir-324	miR-181a*	mir-10a	TPX2	GPR17	PAGE2
mir-760	miR-17-5p	mir-214	CCL16	COL16A1	RNF17
mir-130b	miR-106a	mir-199b	SGOL1	MAB21L1	DDX53
mir-331	miR-301	mir-22	NPY2R	SLC11A1	MAGEB16

## Data Availability

The data presented in this study are openly available at http://acgt.cs.tau.ac.il/multi_omic_benchmark/download.html (accessed on 2 July 2021) as reported by Rappoport et al. [12].

## Supplementary Materials

The following are available online at https://www.mdpi.com/article/10.3390/cancers13143423/s1, Figure S1: Log-rank curve of the four clusters identified by MOUSSE in AML. Figure S2: Log-rank curve of the three clusters identified by MOUSSE in BIC. Figure S3: Log-rank curve of the three clusters identified by MOUSSE in COAD. Figure S4: Log-rank curve of the two clusters identified by MOUSSE in GBM. Figure S5: Log-rank curve of the three clusters identified by MOUSSE in KIRC. Figure S6: Log-rank curve of the four clusters identified by MOUSSE in LIHC. Figure S7: Log-rank curve of the two clusters identified by MOUSSE in LUSC. Figure S8: Log-rank curve of the two clusters identified by MOUSSE in OV. Figure S9: Log-rank curve of the two clusters identified by MOUSSE in SARC. Figure S10: Log-rank curve of the three clusters identified by MOUSSE in SKCM. Figure S11: Plot showing the relative performance (rank) of each tool for each one of the ten cancer datasets. The MOUSSE performance has been highlighted by increasing the thickness of the corresponding line. Table S1: The table includes the references of all the influencing genes extracted in the biological validation. Table S2: The table includes the references of all the relevant miRNAs extracted in the biological validation. Table S3: Signature lengths automatically selected for each cancer in our analysis. Table S4: Kullback–Leibler Divergence between different lengths of the most expressed methylation features (n1) of SKCM. Material S2: Full list of all the significant features extracted for each cancer with Bonferroni corrected p-values and the subject cluster-IDs. Material S3: OpenTargets results with different biomarker lengths.
