# Supplementary material for: MOUSSE: Multi-Omics Using Subject-Specific SignaturEs
Source: Cancers (Basel). 2021 Jul 8;13(14):3423. doi: 10.3390/cancers13143423 (PMC8304726; doi:10.3390/cancers13143423)
Supplement: Supplementary file 1 [file cancers-13-03423-s001.zip › FiorentinoEtAl_SupplementaryData1.pdf]

cancers

Supplementary Materials

MOUSSE: Multi-Omics Using Subject-specific SignaturEs

Giuseppe Fiorentino, Roberto Visintainer, Enrico Domenici, Mario Lauria and Luca Marchetti

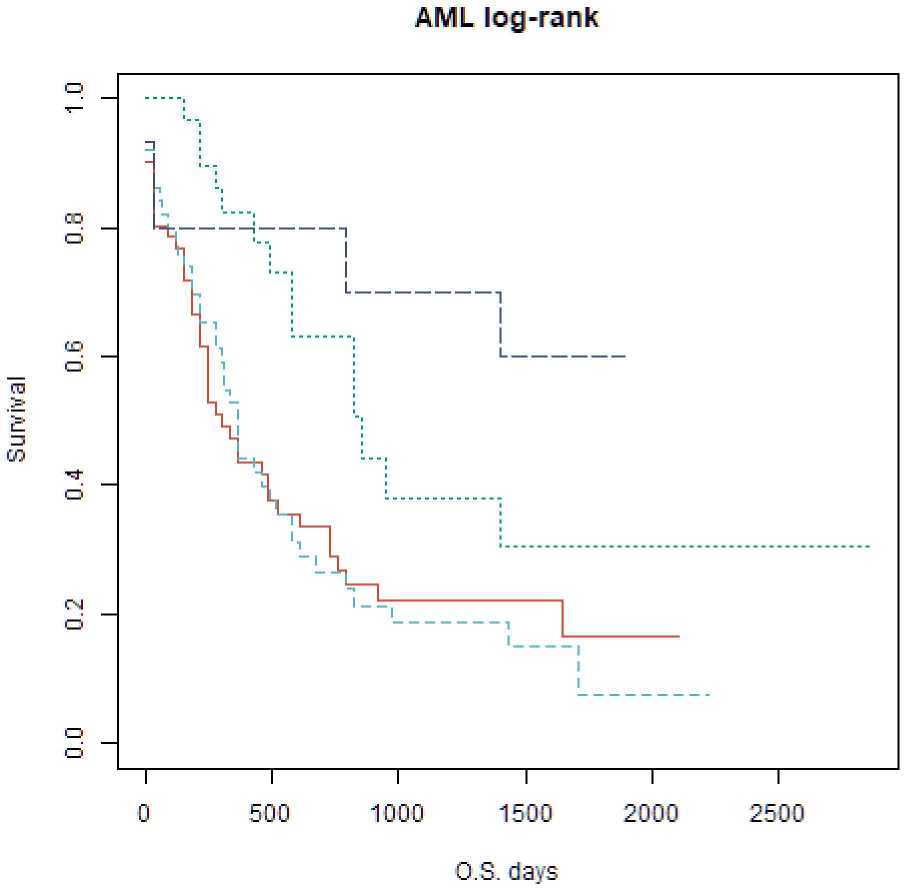

Figure S1 Log-rank curve of the four clusters identified by MOUSSE in AML.

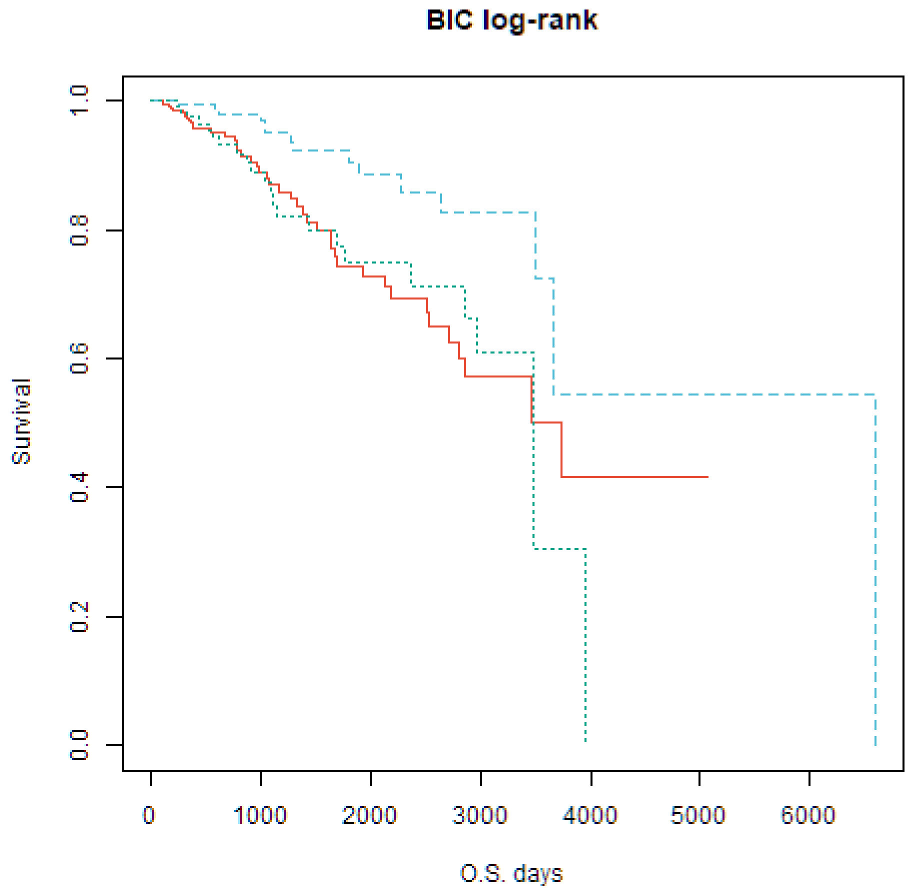

Figure S2 Log-rank curve of the three clusters identified by MOUSSE in BIC.

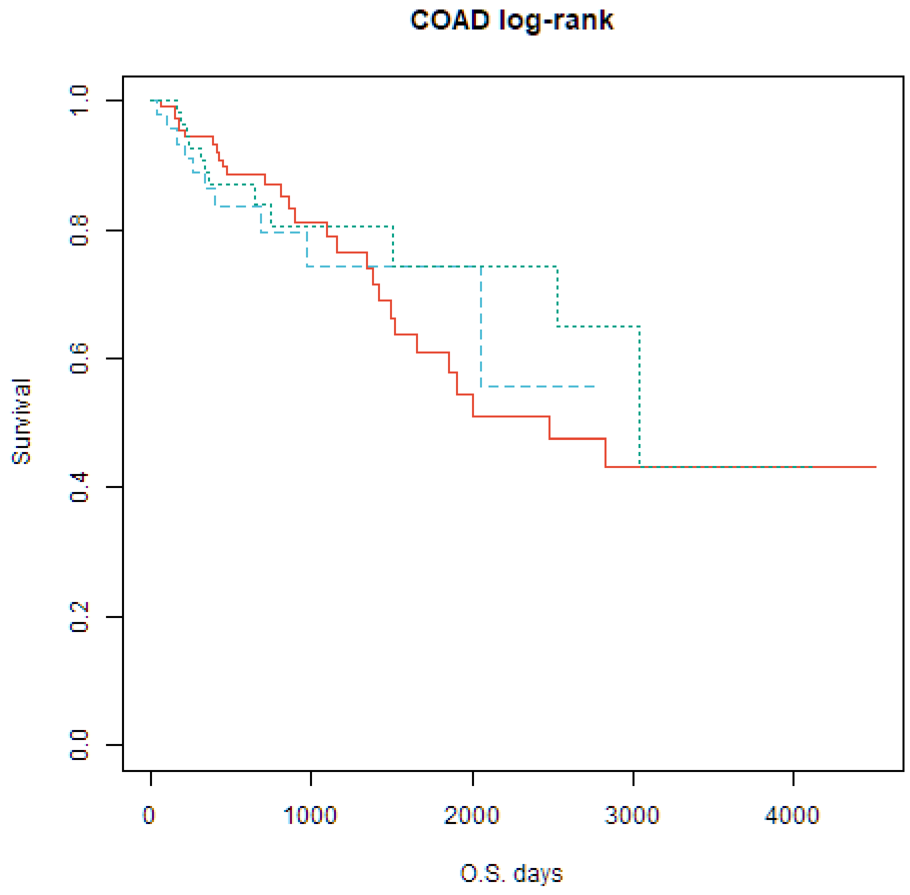

Figure S3 Log-rank curve of the three clusters identified by MOUSSE in COAD.

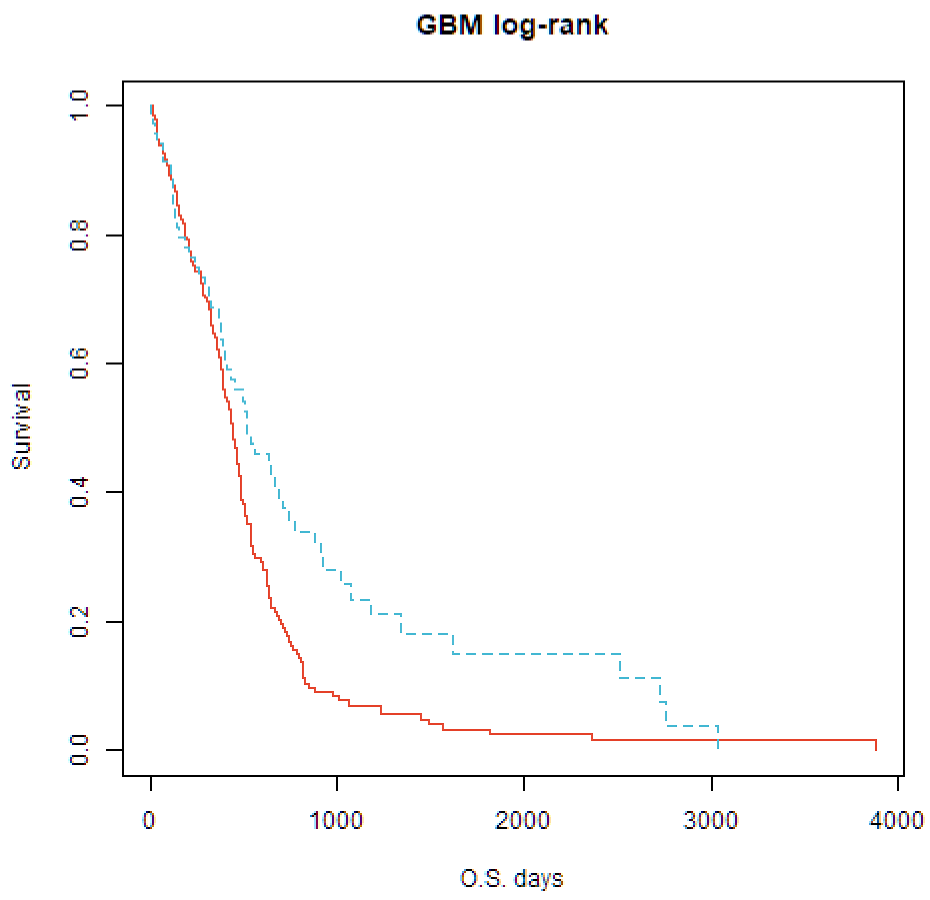

Figure S4 Log-rank curve of the two clusters identified by MOUSSE in GBM.

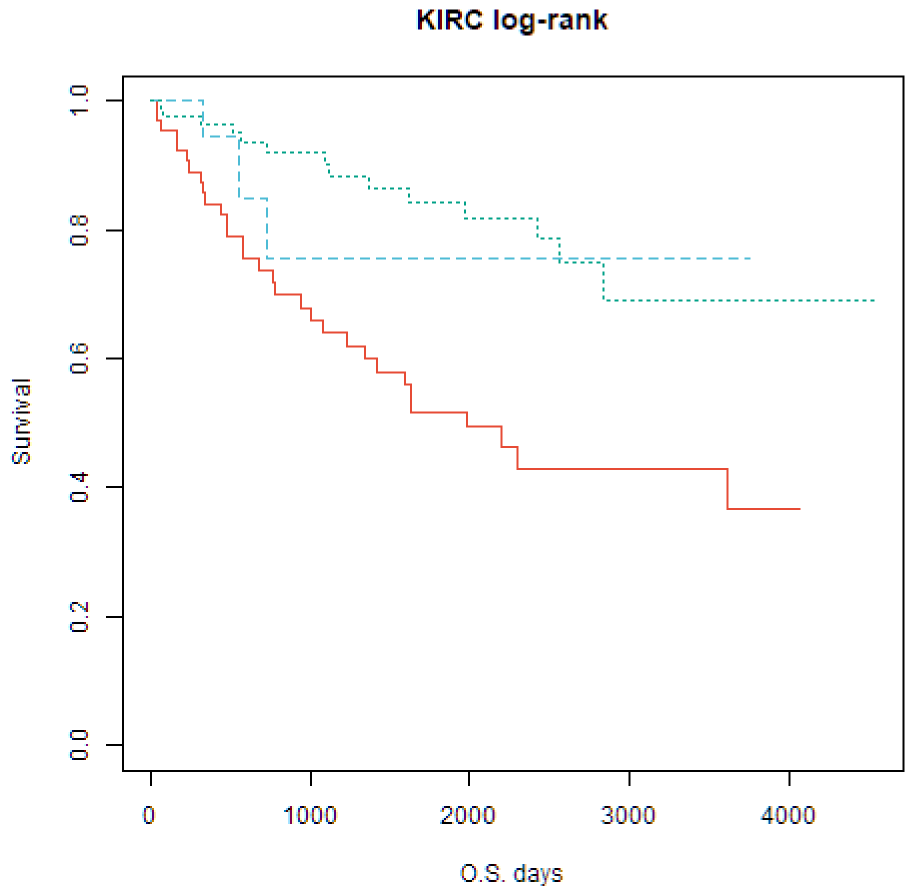

Figure S5 Log-rank curve of the three clusters identified by MOUSSE in KIRC.

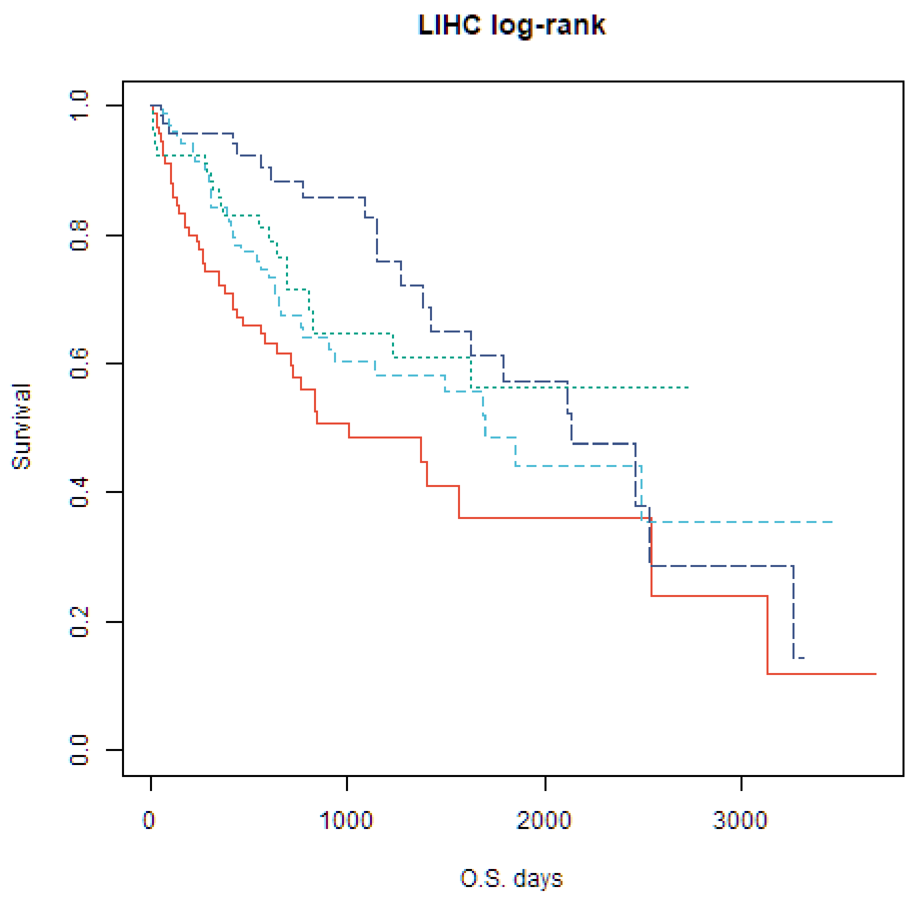

Figure S6 Log-rank curve of the four clusters identified by MOUSSE in LIHC.

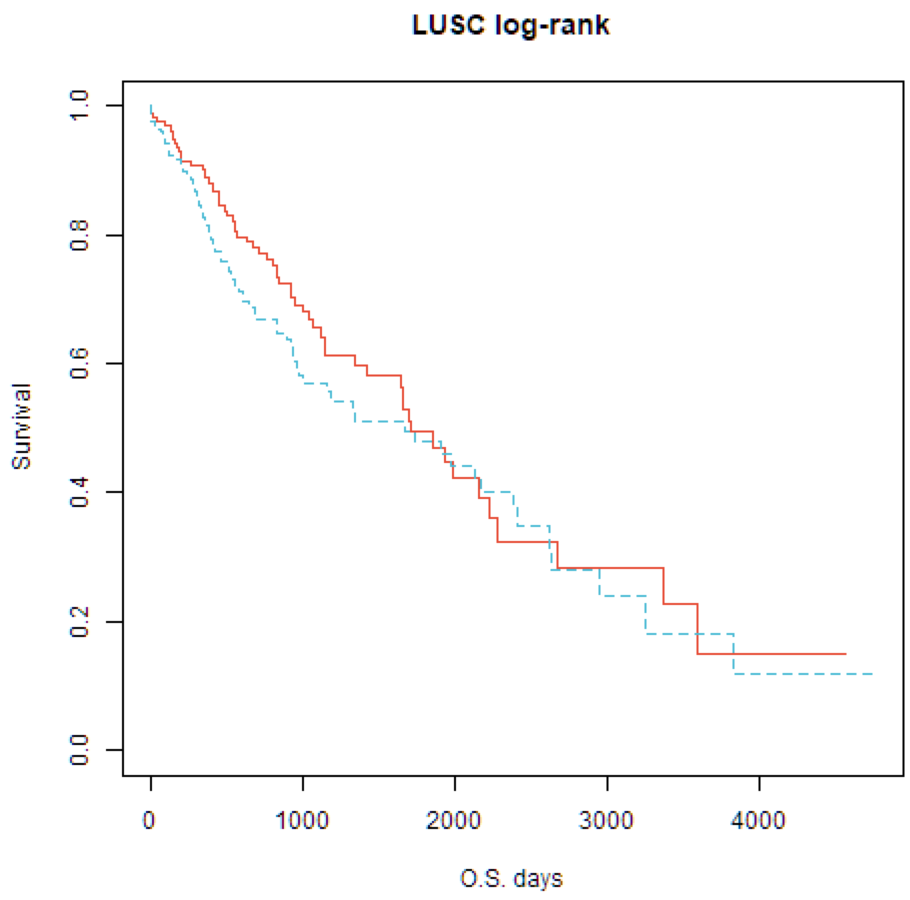

Figure S7 Log-rank curve of the two clusters identified by MOUSSE in LUSC.

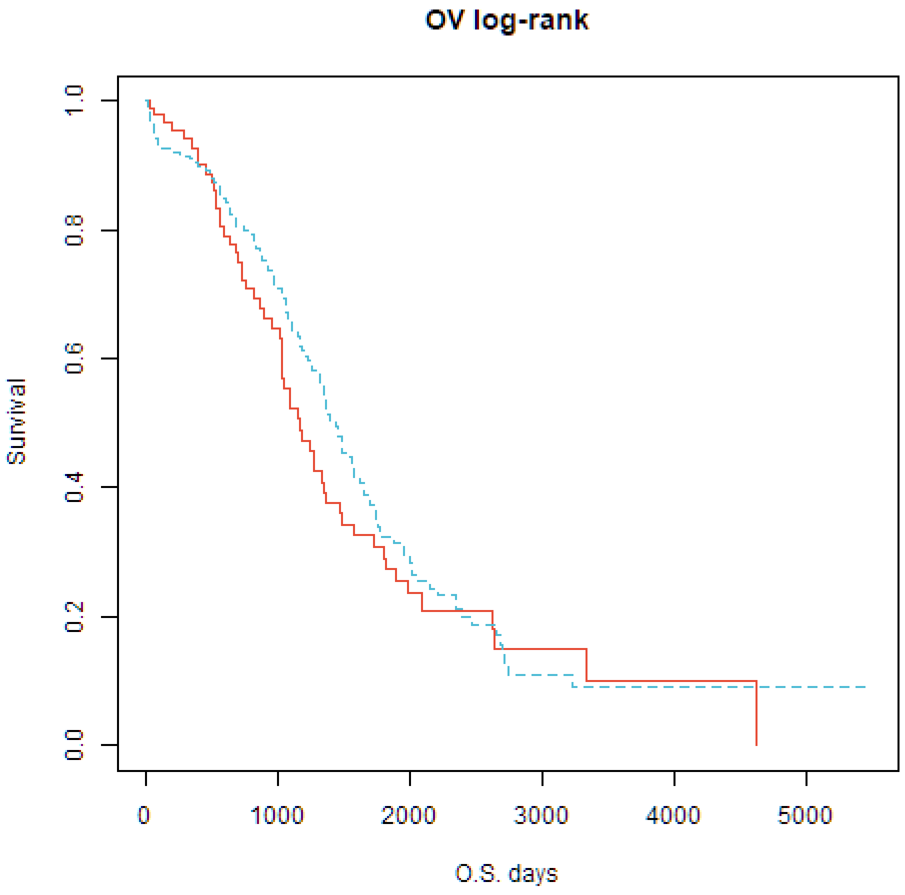

Figure S8 Log-rank curve of the two clusters identified by MOUSSE in OV.

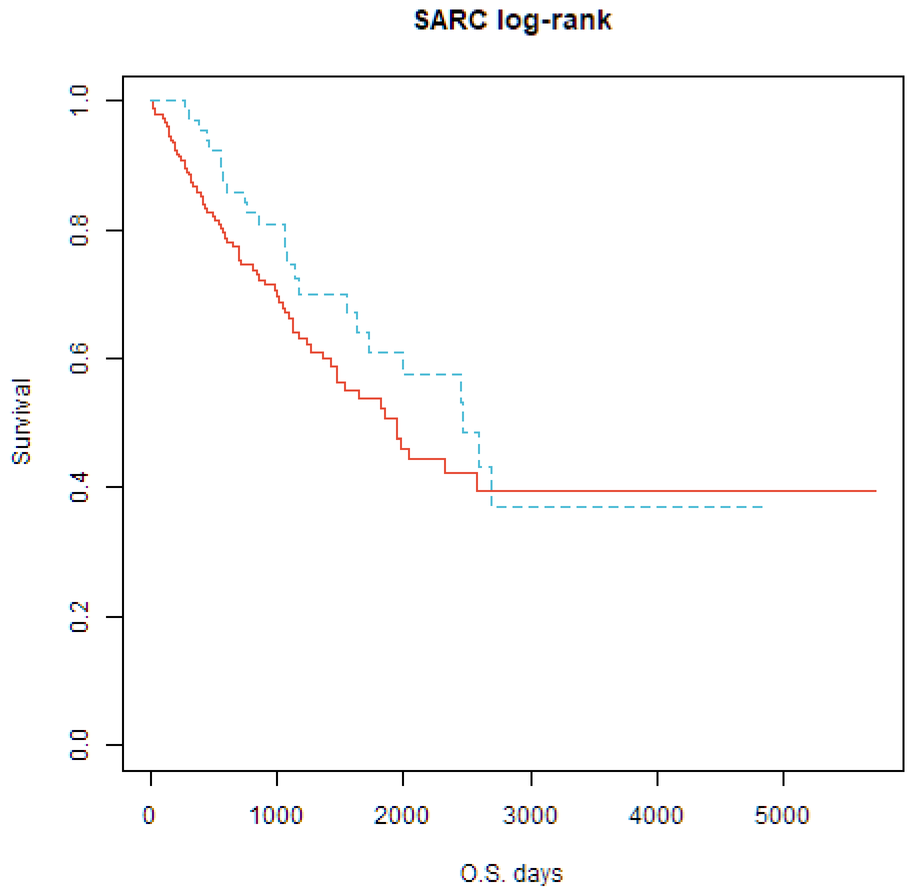

Figure S9 Log-rank curve of the two clusters identified by MOUSSE in SARC.

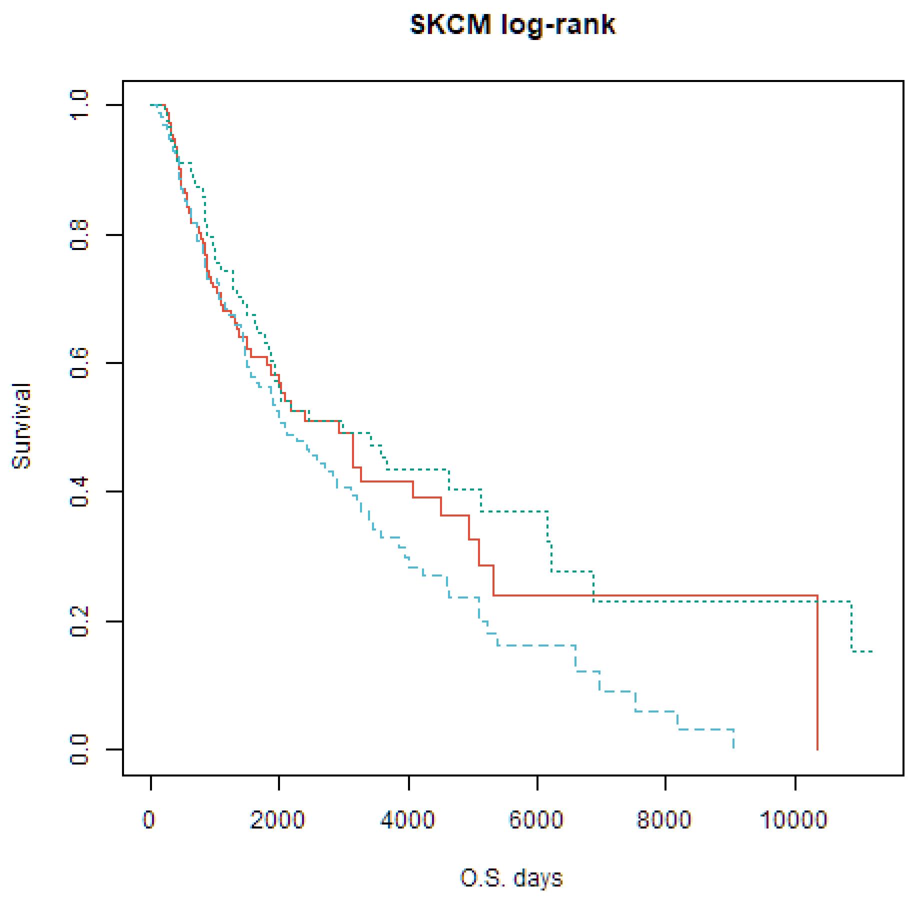

Figure S10 Log-rank curve of the three clusters identified by MOUSSE in SKCM.

Table S1 The table includes the references of all the influencing genes extracted in the biological validation (Table 2). All the references are reported as a brief description of the impact of the gene in the specific cancer and are inserted as hyperlink.

	Genes	Description	DOI
**Breast Cancer (BIC)**	HSPD1	Alters cell proliferation by being regulated by EHMT2	10.3892/ijo.2018.4608
	TPX2	• Promotes migration and invasion.<br>• Knocked down suppresses proliferation and promote apoptosis<br>• Involved in a 4 gene complex influencing breast cancer risk<br>• Used in a 5 genes prognostic biomarker	10.1016/j.bbrc.2018.10.164<br>10.1371/journal.pone.0120020
	SGOL1	An important modulator of centrosome functions in her2+ breast cancer cells	10.1186/1747-1028-9-3
	CCL16	• Enhances T cells reactivity to HER2 cells,<br>• It is an inflammatory chemokine often altered in cancers	
	NPY2R	Differentially expressed in er+/er-	10.1158/1078-0432.CCR-10-1369
**Glioblastoma (GBM)**	SEC61G	Identified as a novel prognostic marker for both survival and therapies (temozolomide, radiotherapy) in GBM	10.12659/MSM.916648
	CRTAC1	• Used in a three-genes prognostic biomarker for lower grade glioma<br>• Copy number variation associated with GBM	10.7717/peerj.8312<br>10.1109/icbbe.2010.5516437
	CA10	Negatively regulate neuronal growth of glioma and its low expression is associated with poor survival	10.1002/1878-0261.12445
	SLC11A1	• Suggested as a prognostic marker, low expression associated with a good prognosis<br>• Its missense mutations occur in GBM	10.18632/oncotarget.24897<br>10.1186/1476-4598-10-17
	PLA2G2A	• One of the most informative features for prognostic deep learning models in GBM	10.3390/cancers11010053
	COL16A1	• Its inhibition reduces glioma cell invasiveness<br>• Causes differential survival in glioma (from proteinatlas)	10.1159/000327947
	GPR17	• Altered expression in glioblastoma<br>• When targeted in murine, leads to the reduction of the neurospheres (a known independent prognostic predictor)	10.1158/0008-5472.CAN-11-2632
	TOX3	• TF regulating neural progenitor identity<br>• Co-expressed with Nesting, a known biomarker for glioma stem cell, can bind Nestin promoters.	10.1016/j.bbagrm.2016.04.005<br>10.5528/wjtm.v4.i3.78<br>10.1016/j.bbrc.2013.03.021
**Liver Hepatocellular Carcinoma (LIHC)**	EXO1	Overexpression associated with poor prognosis	10.1080/15384101.2018.1534511
	NEK2	• Promotes migration and invasion, linked with poor prognosis<br>• Influences sorafenib resistance (unknown how)	10.3892/or.2018.6224<br>10.1186/s13046-019-1311-z
	RNF17	Differentially expressed and used in a prognostic model of RNA binding proteins	10.21203/rs.3.rs-40802/v2
	DDX53	• Expressed in a variety of cancers, especially in testis	10.1006/bbrc.2002.6701

		• Confers drugs resistance by regulating p53<br>• Seen in aberrant expression in other cancers	10.1074/jbc.M109.095950<br>10.1016/s0006-291x(03)01121-5
	DSCR4	Identified as differentially expressed between normal tissue and primary tumor	10.2147/CMAR.S186561

Table S2 The table includes the references of all the relevant miRNAs extracted in the biological validation (Table 2). All the references are reported as a brief description of the impact of the gene in the specific cancer and are inserted as hyperlink.

	miRNAs	Description	
Breast Cancer (BIC)	let-7c	High expression leads to better prognosis in ER+, blocking estrogen-activated Wnt signaling in induction of self-renewal	10.1038/cgt.2016.3
	mir-140	Downregulation promotes cancer stem cell formation	10.1038/onc.2013.226
	mir-1307	In a miRNAs signature used to predict BC stage	10.1038/s41598-018-34604-3
	mir-33b	Inhibits metastasis by targeting HMGA2, SALL4 and Twist1	10.1038/srep09995
	mir-324	When sponged by LINC00963, they promote tumorigenesis and radioresistance	10.1016/j.omtn.2019.09.033
	mir-760	• Influences (Doxorubicin/ Nanog) chemoresistance<br>• Considered a potential biomarker for cancer detection	10.1016/j.biopha.2014.11.028<br>10.1016/j.gene.2020.144648
	mir-130b	• Targets PTEN to mediate drug resistance and proliferation of BC cells<br>• Inhibits cell invasion and migration by targeting DLL1 on<br>• Deregulated in triple negative BC, represses CCNG2	10.1038/srep41942<br>10.1016/j.gene.2017.01.036<br>10.1186/s12943-015-0301-9
	mir-331	• Significantly over-expressed in metastatic BC, can be used to differentiate from luminal A<br>• Overexpression linked to poor prognosis, promotes progression<br>• Promotes cell proliferation by targeting SRCIN1<br>• Essential for HER2+ cell growth	10.1186/s12885-019-5636-y<br>10.1159/000508792<br>10.1016/j.molonc.2013.10.001
Glioblastoma(GBM)	miR-222	• Linked to cell lysis and proliferation in GBM<br>• Overexpressed with mir-221 increase GBM invasiveness by targeting PTPμ, expression is correlated with glioma grade<br>• High level increase cell invasion leading to poor prognosis (with 221)<br>• Targeting mir-221/222 induces apoptosis in glioma cell lines	10.1177/1947601912448068<br>10.1038/onc.2011.280<br>10.3892/ijo.2015.3308<br>10.1186/1479-5876-10-119<br>10.1007/s12035-014-9079-9

		• Descriptive and prognostic biomarker	
	miR-23a	• Promotes invasion of GBM<br>• H Can be targeted via HOXD10 to inhibit cell invasion<br>• Upregulated in glioma, anti 23a suppresses glioma cell growth by targeting APAF1	10.1038/s41392-018-0033-6<br>10.2174/138161281131999905<br>09 10.1038/srep03423
	miR-204	• Supresses GBM progression by targeting atf2<br>• Act as tumour suppressor gene partly by suppressing CYP27A1<br>• Upregulated by xanthohumol, targets the IGFBP2 pathway to induce apoptosis in glioma cells<br>• Characterize an aggressive subset of medulloblastomas	10.18632/oncotarget.11732<br>10.3892/ol.2018.8846<br>10.1016/j.neuropharm.2016.0<br>7.038<br>10.1186/s40478-019-0697-3
**Glioblastoma (GBM)**	miR-221	• Linked to cell lysis and proliferation in GBM<br>• Overexpressed with mir-222 increase GBM invasiveness by targeting PTPμ, expression is correlated with glioma grades<br>• High-level increase cell invasion leading to poor prognosis (with 222)<br>• Targeting mir-221/222 induces apoptosis in glioma cell lines<br>• Descriptive and prognostic biomarker	10.1177/1947601912448068<br>10.1038/onc.2011.280<br>10.3892/ijo.2015.3308<br>10.1186/1479-5876-10-119<br>10.1007/s12035-014-9079-9
	miR-340	• Suppresses GBM, high expression linked with good prognosis<br>• Normally downregulated in GBM, inhibits cell proliferation by targeting CDK6, cyclin-D1, and cyclin-D2	10.18632/oncotarget.3288<br>10.1016/j.bbrc.2015.03.088
	miR-181a*	• Downregulated in GBM, indirectly correlated with glioma grade, transfected reduce cell proliferation, invasion and increases apoptosis and radiosensitivity<br>• Predict response to concomitant chemo/radiotherapy with temozolomide	10.1177/1947601912448068<br>10.4149/neo_2010_03_264
	miR-17-5p	• Low levels are positively associated with advanced clinical stage, incidence of relapse, and tumour differentiation. Highly reduced post radiotherapy can inhibit autophagy.<br>• Can repress MDM2, resulting in decreased cell proliferation and drug resistance.	10.3727/096504016X14719078<br>133285<br>10.18632/oncotarget.810<br>10.1177/1947601912448068
	miR-106a	• Inhibits cell growth by targeting E2F1 (independently by p53), a low expression associated to high-grade glioma<br>• Independent Prognostic marker	10.1177/1947601912448068<br>10.1007/s00109-011-0775-x<br>10.1093/NEUONC/NOT001

	miR-301	• Serum exosomal mir-301a is considered a diagnostic and prognostic biomarker<br>• Differentially expressed in glioblastoma stem-like cells, upregulation leads to poor prognosis	10.1007/s13402-017-0355-3<br>10.1038/s41598-018-20929-6
Liver Hepatocellular Carcinoma (LIHC)	mir-105-2<br>mir-767<br>mir-105-1	Together in a cluster, upregulation linked to poor prognosis and resistance of sorafenib, all three miRNAs are considered independent prognostic factors	10.1186/s40364-020-0186-7
	mir-139	• Low expression results in poor outcome<br>• Inhibits cell growth<br>• Suppresses metastasis	10.3892/ol.2019.11031<br>10.1186/s13046-019-1175-2<br>10.1053/j.gastro.2010.10.006
	mir-199a-1<br>mir-199a-2	• Low expression associated with poor survival, overexpression inhibits cell proliferation migration and invasion<br>• Transfused increase chemosensitivity<br>• Used in a prognosis biomarker signature in Egyptian patients	10.18632/oncotarget.18052<br>10.1186/s13046-019-1512-5<br>10.1007/s40291-015-0148-1
	mir-214	• Inhibits proliferation and migration targeting foxm1<br>• Effect on proapoptotic and anti-angiogenic genes	10.1038/s41434-018-0029-4<br>10.1007/s12291-019-00824-1
	mir-199b	• Downregulation predicts poor outcomes, overexpression promotes cells aggregation, suppresses cell migration and HCC invasivity.	10.1038/bjc.2017.164
	mir-22	• Downregulated in HCC, low expression correlated with prognosis, lower cell proliferation ,<br>• Correlated with ezrin, a protein already associated with clinical outcome<br>• Expression inversely correlated with metastatic ability, interacts with CD147	10.1038/sj.bjc.6605895<br>10.1177/0300060513484436<br>10.1186/s12935-016-0380-8

Table S3 Signature lengths automatically selected for each cancer in our analysis. Top/Bot indicate the lengths used respectively for the most (top) and least (bot) expressed features.

	AML	BIC	COAD	GBM	KIRC	LIHC	LUSC	OV	SARC	SKCM
EXP-Top	250	250	250	250	250	250	250	250	250	250
EXP-Bot	250	250	250	250	250	250	250	250	250	250
MET-Top	150	100	100	150	150	250	100	100	100	150
MET-Bot	100	100	150	100	100	200	100	100	100	100
miRNA-To	250	100	100	100	100	150	100	100	100	150
miRNA-Bo	250	100	100	100	250	100	100	250	100	100

Table S4 Kullback- Leibler Divergence between different lengths of the most expressed methylation features (n1) of SKCM. The divergence achieved its maximum at the length of 150.

**SKCM (n1)**	**25**	**50**	**100**	**150**	**200**	**250**
**25**	0	1.742154	2.476254	**2.529776**	2.463618	2.307688
**50**	1.742154	0	0.842672	0.956191	0.935043	0.902527
**100**	2.476254	0.842672	0	0.246036	0.305915	0.312584
**150**	**2.529776**	0.956191	0.246036	0	0.088021	0.150315
**200**	2.463618	0.935043	0.305915	0.088021	0	0.025236
**250**	2.307688	0.902527	0.312584	0.150315	0.025236	0

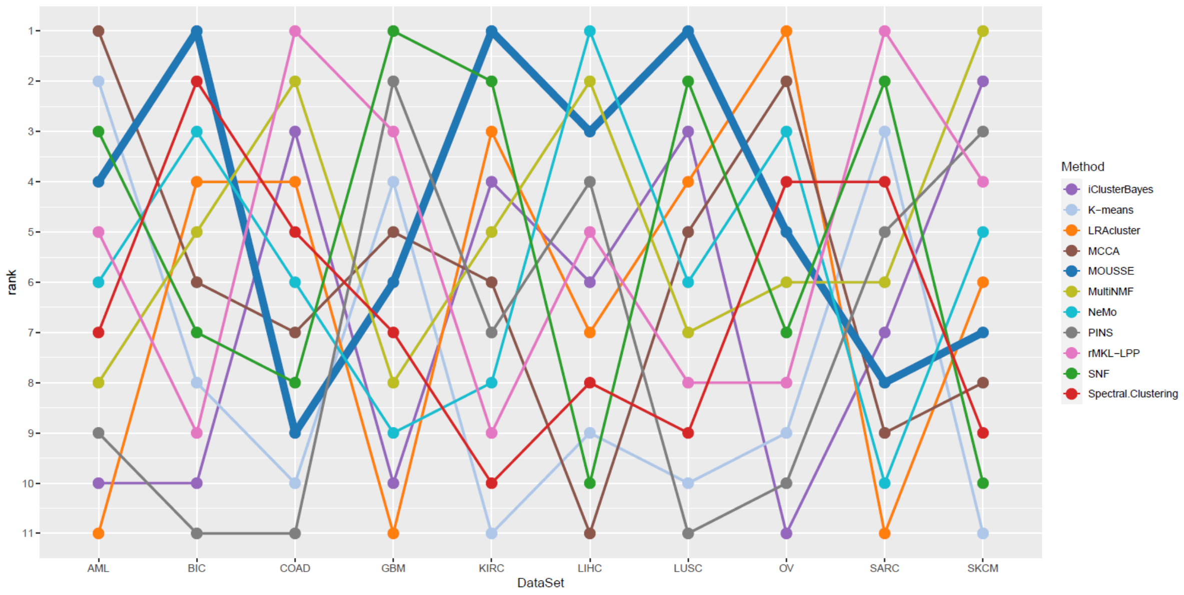

Figure S11 Plot showing the relative performance (rank) of each tool for each one of the ten cancer datasets. The MOUSSE performance has been highlighted by increasing the thickness of the corresponding line.

Detailed description of the pipeline.

Here we give a more detailed description of the pipeline in the form of pseudocode to increase result reproducibility.

Input: N omics datasets (in our test N=3), each dataset is a matrix (*m* x *n*), where the columns, *n*, are the subjects and the rows, *m,* are the features of the omics (*e.g.*, genes, miRNAs *etc.*). It is assumed that the *n* subjects are the same in all the datasets (multi-omics dataset).
As reported in Materials and Methods, section 4.1, all the data required to reproduce our analysis are available for download from Shamir's lab website: http://acgt.cs.tau.ac.il/multi_omic_benchmark/download.html .
Output: A partitioning of the subjects in clusters based on their similarities in the multi-omics network.

Procedures repeated for each omics
Preprocessing (section 4.2.1):

1. In Rappoport work, both gene expression and miRNAs were converted into logarithm scale and underwent a light pre-processing as follows:
    a. Log transform gene and miRNA values.
    b. For each feature *f* in a dataset, compute the mean and standard deviation across all subjects.
    c. Remove features with a standard deviation equal to zero.
    d. Normalize the values of the *m*-th feature by subtracting the mean and by dividing for the standard deviation.
2. Feature filtering:
    a. Produce a vector containing the coefficient of variation (CV) of every feature.
    b. Remove all the features with a CV lower than a user-provided threshold (in our analysis 5th percentile of the CVs computed at step 2.a).

Subject-Specific Signature Extraction (section 4.2.2):

3. Sort all the features from highest to lowest value, separately for each subject.
4. Create a matrix of the same size of the dataset containing the feature IDs according to the order computed in the previous step.
5. Extract the n_1 top features and the n_2 bottom features for each subject, obtaining a set of two matrices of size (n_1 x *n*) and (n_2 x *n*) of feature IDs, called top and bottom matrices, respectively. The n_1 and n_2 values are user-provided, for those used in our analysis please refer to Supplementary Table S3.
6. Reverse the row order of the bottom matrix so that the lowest features will have the highest rank.

Omics-specific similarity networks (section 4.2.3):

7. Set the RBO parameters:
    a. Select a weight (W_{RBO}) to give the desired relevance to the upper part of a sorted list. In our analysis, we set W_{RBO} = 0.8, as suggested in (Webber *et al.* 2010).
    b. Find the value of the parameter p that satisfies the following formula:

    $$W_{RBO} = 1 - p^{d-1} + \frac{1-p}{p} \cdot d \cdot (ln\frac{1}{1-p} - \sum_{i=1}^{d-1}\frac{p^i}{i})$$

    where d is, in turn, the signature length n_1 or n_2.
8. Compute the RBO between each subject pair, separately for each of the omics' matrices (top and bottom).
9. Store the RBO computed similarity values in two matrices of size (n x n).

Signature Length Optimization (section 4.2.4):

Optionally a routine for the optimization of signature length can be used. This routine essentially tests a vector of different user-provided values for n_1 and n_2, by iteratively executing steps 3 to 9 for each value. This will produce multiple similarity matrices of the same omics that will be compared using the Kullback-Leibler divergence. To do so:

10. For each similarity matrix, produce the distribution of the similarity values. In our analysis, the bin width for the distribution was set to 0.001
11. Add a pseudo-count of 1 to each bin in order to avoid bin values of 0 and rescale the distribution to obtain a unitary area.
12. Compute the Kullback-Leibler divergence between the distribution associated with the shortest signature and those of all the other lengths.
13. Select the signature length associated with the maximum Kullback-Leibler divergence.

Procedure for merging the omics-specific networks.

Network integration and Clustering (section 4.2.5):

14. For each omics, fuse the top and bottom matrices into one by calculating the mean of the two similarity matrices.
15. Fuse the resulting omics networks using the SNF function of the "SNFtools" package. In our analysis, we set the required SNF parameters K (number of neighbors) and T (iterations in the diffusion process) respectively to one-tenth of the number of subjects and to 30 iterations.
16. Cluster the subjects in the resulting integrated network using a user-provided graph clustering function. For our analysis, we used the SpectralClustering function in the "SNFtools" package. We used the "estimateNumberOfClustersGivenGraph" function in the same package to estimate the number of clusters, using the best rotation cost option.

Additional procedure

Here we add a procedure to perform the cluster validation used in our analysis. This was written using TCGA data in mind and it might require modifications for other datasets.

Survival Analysis (section 4.3):

To verify the clustering performance, we performed a survival analysis between the identified clusters.

17. Produce a matrix with all the subjects Overall Survival Time, Overall Survival, and cluster-ID.
18. Extract a chi-squared of the difference in survival between the subjects belonging to the identified clusters using the SurvDiff function of the "survival" R package.
19. Calculate a p-value subtracting from 1 the output of the "pchisq" R function, with the number of clusters minus one as degrees of freedom.
20. Randomize the clusters labels and repeat step 19 on the new clusters.
21. Repeat the procedure in step 20 for 3000 times to obtain an empirical distribution of p-values.
22. Calculate a classification score by comparing the result obtained in step 19 against the empirical distribution.
23. Report the final classification score as the negative logarithm in base ten of the resulting score.
